# The Present and Future of Clinical Management in Metastatic Breast Cancer

**DOI:** 10.3390/jcm11195891

**Published:** 2022-10-05

**Authors:** Pauline H. Lin, George Laliotis

**Affiliations:** 1 Department of Internal Medicine, Advocate Christ Medical Center, Oak Lawn, IL 60453, USA; 2 Sidney Kimmel Comprehensive Cancer Center, Department of Oncology, Johns Hopkins School of Medicine, Baltimore, MD 21205, USA

**Keywords:** breast, cancer, metastasis, oncology

## Abstract

Regardless of the advances in our ability to detect early and treat breast cancer, it is still one of the common types of malignancy worldwide, with the majority of patients decease upon metastatic disease. Nevertheless, due to these advances, we have extensively characterized the drivers and molecular profiling of breast cancer and further dividing it into subtypes. These subgroups are based on immunohistological markers (Estrogen Receptor-ER; Progesterone Receptor-PR and Human Epidermal Growth Factor Receptor 2-HER-2) and transcriptomic signatures with distinct therapeutic approaches and regiments. These therapeutic approaches include targeted therapy (HER-2^+^), endocrine therapy (HR^+^) or chemotherapy (TNBC) with optional combination radiotherapy, depending on clinical stage. Technological and scientific advances in the identification of molecular pathways that contribute to therapy-resistance and establishment of metastatic disease, have provided the rationale for revolutionary targeted approaches against Cyclin-Dependent Kinases 4/6 (CDK4/6), PI3 Kinase (PI3K), Poly ADP Ribose Polymerase (PARP) and Programmed Death-Ligand 1 (PD-L1), among others. In this review, we focus on the comprehensive overview of epidemiology and current standard of care treatment of metastatic breast cancer, along with ongoing clinical trials. Towards this goal, we utilized available literature from PubMed and ongoing clinical trial information from clinicaltrials.gov to reflect the up to date and future treatment options for metastatic breast cancer.

## 1. Introduction

Breast cancer is the leading cause of cancer globally in women [1,2] with approximately 2.3 million new cases worldwide, contributing to almost 12% of all cancer cases [1,3]. According to the GLOBOCAN estimates of cancer incidence and mortality, breast cancer accounts for 1 in 4 cancer cases in women, which comprises the majority of incidence in the large majority of countries [1]. A recent population-based study in countries with low to medium income, identified a greater incident of both premenopausal and postmenopausal breast cancer with increasing case fatalities, attributing to growing inequities to affordable and standard of care-quality treatment [4].

Despite the recent advances in treatment, follow-up and targeted therapies, around 30% of breast cancer patients still eventually relapse with distant metastasis [5], which develops approximately 5–20 years after the initial diagnosis [6]. It is worth mentioning that this relapse interval in this disease is largely dependent on the molecular subtype, with Triple-Negative Breast Cancer, to relapse and/or metastasize sooner compared to other subtypes [6]. Metastatic disease remains the most common cause of death in 90% of the patients with breast cancer [7,8]. The recent data regarding the efficacy of PI3-Kinase [9,10] and PARP [11,12] inhibitors in metastatic breast cancer (mBC) treatment, identified the importance of clinicians to be familiar with the recent advancements in experimental clinical and basic research. Here, we review the clinical and molecular subtypes of breast cancer and the organotropism of their metastatic pattern, the epidemiology and predictive/prognostic factors, the standard-of-care treatment options and the current advancements in clinical trials in the management of metastatic breast cancer.

## 2. Molecular Classification of Breast Cancer

**Figure 1:**
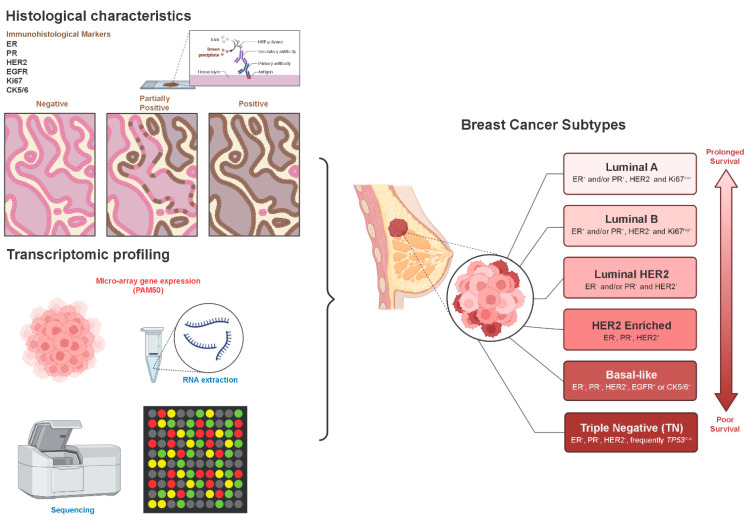
Methods of subclassification and subtypes of breast cancer. The molecular subtypes of breast cancer are classified based on histological and molecular markers that are identified by Immunohistochemistry (IHC) and microarrays-based techniques, respectively. Based on the combination of these markers, breast cancer patients can be stratified in 6 subtypes with different median survival upon the diagnosis of the distant metastatic disease. Abbreviations: ER: Estrogen Receptor, PR: Progesterone Receptor, HER2: Human Epidermal Growth Factor Receptor 2, EGFR: Epidermal Growth Factor Receptor, Ki67: Marker of Proliferation Ki-67, CK5/6: Cytokeratin 5/6.

Breast cancer is an intrinsically heterogeneous and complex disease with various molecular subtypes, histological features, and clinical characteristics [2,13]. These markers are analyzed by Immunohistochemistry (IHC) or gene expression assays (PAM50 micro-array markers) and include the Hormone Receptors (HR), Estrogen Receptor (ER), Progesterone Receptor (PR), Human Epidermal Growth Factor Receptor 2 (HER-2), the cell proliferation marker Ki67, cytokeratin 5/6 (CK5/6), and Epidermal Growth Factor Receptor (EGFR) [13,14,15]. Based on these markers, breast cancer can be classified in luminal A (ER^+^ and/or PR^+^, HER-2^−^ and Ki67^low^), luminal B (ER^+^ and/or PR^+^, HER-2^−^ and Ki67^high^), luminal-HER-2 (ER^+^ and/or PR^+^, and HER-2^+^), HER2-enriched (ER^−^, PR^−^, HER-2^+^), basal-like (ER^−^, PR^−^, HER-2^−^, and EFGR^+^ or CK5/6^+^), and triple-negative phenotype (TNBC) (ER^−^, PR^−^, HER-2^−^) [13,14,15] (Figure 1). It is important to note that the TNBC frequently harbors *TP53* mutations and 80% of them express basal-like markers [10, 13,14,15].

## 3. Epidemiology and Predictive/Prognostic Factors of mBC

The overall prevalence of mBC, which includes de novo mBC (dn mBC) and recurrent mBC, has not been widely studied due to lack of an organized US population-based registry. Utilizing data from the US Surveillance Epidemiology and End Results (SEER) Program, a recent analysis estimated that in 2013, 138,622 of patients were living with mBC, while 28% (38,897 of 138,622) of them had presented with dn mBC [16,17]. It is worth mentioning the higher frequency of dnMBC in low- and middle-income countries compared to high-income, most likely due to limited access to both screening and standard-of-care treatments, identifying another public health perspective in the disparities of the management of cancer patients [18]. Specifically, compared to Northern Europe and Northern America, where the Age Standardized Rate (ASR per 100,000 women) is 90.1 and 84.8, respectively, the incidence rate in low- and middle-income regions, such as Eastern and Western Africa, South Central Asia and Micronesia are 39.2, 37.3, 25.9 and 58.2, respectively [19].

**Figure 2:**
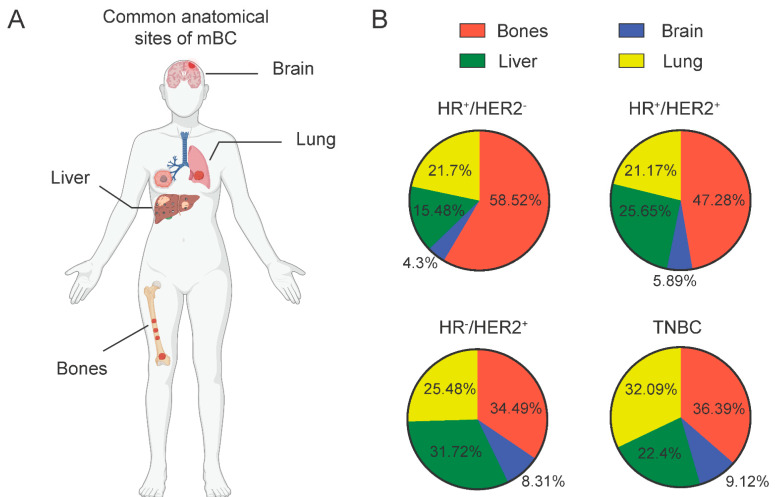
Common metastatic sites of breast cancer subtypes. (**A**) Representation of common metastatic sites in breast cancer patients. (**B**) The frequencies of metastatic sites in breast cancer subtypes, as described previously [22], represented as pie charts.

Breast cancer most commonly metastasizes in anatomical sites which include the bone, brain, liver, and lung [6]. This process involves a cascade initiated by local invasion and migration through stromal connective tissues, sequenced by intravasation into the blood and lymphatic vessels, leading to extravasation and infiltration into the tissue parenchyma of the secondary organ site [20]. A metastatic spread involves multiple factors, one of which is the molecular subtypes which are greatly associated with the increased risk of spread to a specific site [21]. In a recent study [21], it was found that HR^+^ cancers are found to have increased frequency of metastasis to the bones, among other subtypes. On the other hand, HER-2^+^ and TNBC subtypes are associated with higher prevalence of brain metastasis [22]. In a similar fashion, although lung and bone metastases can occur in all breast cancer subtypes, they are more often associated with HR^+^ cases, while liver metastasis with HER-2^+^ subtypes [21,22] (Figure 2). Intrinsic molecular and genomic characteristics have been linked with this organotropism of metastatic breast cancer [23], a topic that goes beyond the purpose of this review.

Given the complexity and the poor outcomes of patients with mBC [24], it is important to acknowledge the prognostic and predictive factors of metastatic disease, in order to stratify patients in higher and lower risk and to aid the selection of specific therapies. Prognostic factors provide information on clinical outcome at the time of diagnosis or patient course with metastatic disease, independent of therapeutic approach. The most common and useful prognostic factors are usually clinical variables [25]. By contrast, predictive factors provide information on the likelihood of response to a given therapy [26,27,28]. Table 1 shows all the available prognostic and predictive factors to date.

## 4. Current Treatment Options of mBC

As analyzed above, breast cancer subtypes and classifications are well-characterized and personalized for each patient group. To this extent, given the distinct classification of breast cancer, the therapeutic decision and algorithms of metastatic disease is largely dependent on its molecular subclassification and on HR and HER-2 expression status.

### 4.1. Treatment of Hormone Receptor Positive mBC

The treatment of HR^+^ mBC is defined by numerous clinical factors. These factors include the menopausal status (pre- or post-) at the time of metastatic disease, the recurrence of metastatic disease, the time interval between each recurrence episode, the status of specific concurrent mutations (e.g., *PIK3CA* and *BRCA* mutations), the presence of bone or visceral metastatic disease and the overall performance status. It is also worth mentioning that in clinical practice, de novo metastatic disease, recurrence after more than 12 months of adjuvant therapy and bone metastasis, fall into the endocrine-sensitive subgroups of patients [40]. Lastly, it is important to note that clinicians should obtain clinical tumor samples at baseline and at the treatment naive stage, since the therapeutic decisions depend on Next Generation Sequencing, transcriptomic and mutational characteristics of the tumor. This allows us to compare the biological development of the early stage versus the metastatic tumor, to better guide clinical decisions [41].

**Figure 3:**
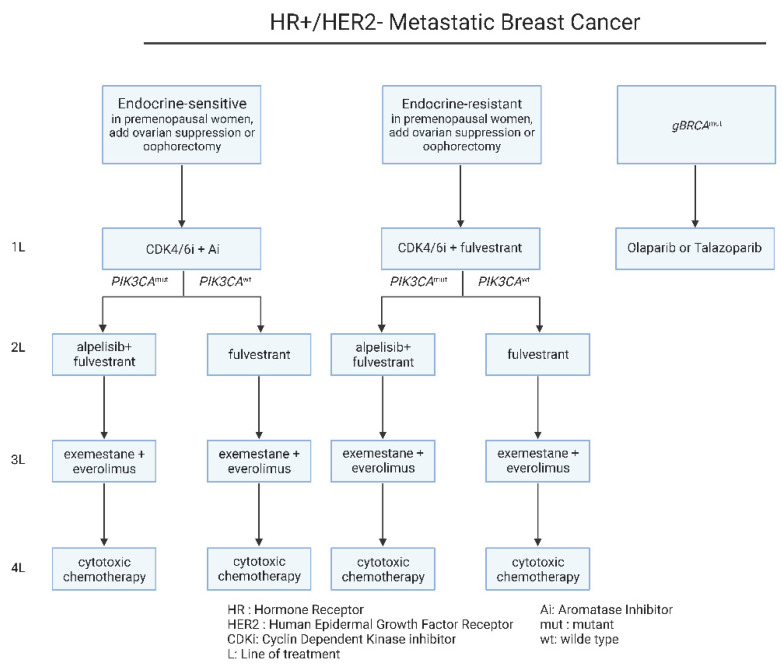
Current therapeutic algorithm for the management of HR^+^/HER-2^−^ mBC. Proposed therapeutic algorithm for patients with HR^+^/HER-2^−^ metastatic breast cancer [39]. The abbreviations of the terms used in the figure are outlined in the lower part of the algorithm.

The main clinical first line recommendation depends on the recurrence time interval and the menopausal status (Figure 3). In estrogen-sensitive cases, the administration of CDK4/6 with an aromatase inhibitor, should be considered the standard-of-care option in these patients [42,43]. CDK4/6 inhibitors have been approved more than 6 years ago for metastatic ER^+^ metastatic disease, based on the findings of PALOMA-1 trial [43]. Furthermore, the combination of CDK4/6i, ribociclib plus estrogen therapy significantly improved overall survival (OS) relative to estrogen therapy alone, according to the important phase III MONALEESA-2, MONALEESA-3, and MONALEESA-7 trials [44]. On the other hand, regarding the estrogen-resistant cases or in cases with no suitability for aromatase inhibitors, CDK4/6 inhibitors should be combined with fulvestrant, an estrogen degrader [45,46,47].

Following disease progression upon first-line treatment, in the case of the estrogen-resistant groups, *PIK3CA* mutational status defines the therapeutic decisions. In patients harboring *PIK3CA* mutations, fulvestrant can be combined with alpelisib, a PIK3α specific inhibitor [48]. Alpelisib has been approved as a combination therapy with fulvestrant for *PIK3CA* mutated ER^+^/HER-2^−^ metastatic breast cancer, upon the findings of SOLAR-1 clinical trial [9]. On the other hand, the estrogen-sensitive patients with recurrence on CDK4/6 inhibitors, can be treated with an aromatase inhibitor in combination with the mTOR inhibitor, everolimus [48] (Figure 3). Beyond these therapeutic strategies, subsequent lines of therapy include cytotoxic chemotherapy for all patients [49,50,51] (Figure 3). On a different note, the administration of the same chemotherapeutic regimen upon recurrence, is not recommended, with the exemption of taxanes that can be used upon early and metastatic disease [40,49,50].

### 4.2. Treatment of HER-2 Positive mBC

Traditionally, the HER-2^+^ breast cancer has been a more aggressive clinical subtype compared to the HR^+^ subtype, with poorer clinical outcomes [29,52]. Nevertheless, due to advancements in drug development and introduction of HER-2 targeting therapies, such as trastuzumab and trastuzumab-emtansine (T-DM1), the median survival of these patients has been increased to 5 years, and up to 8 years in 30–40% of the cases [52,53].

**Figure 4:**
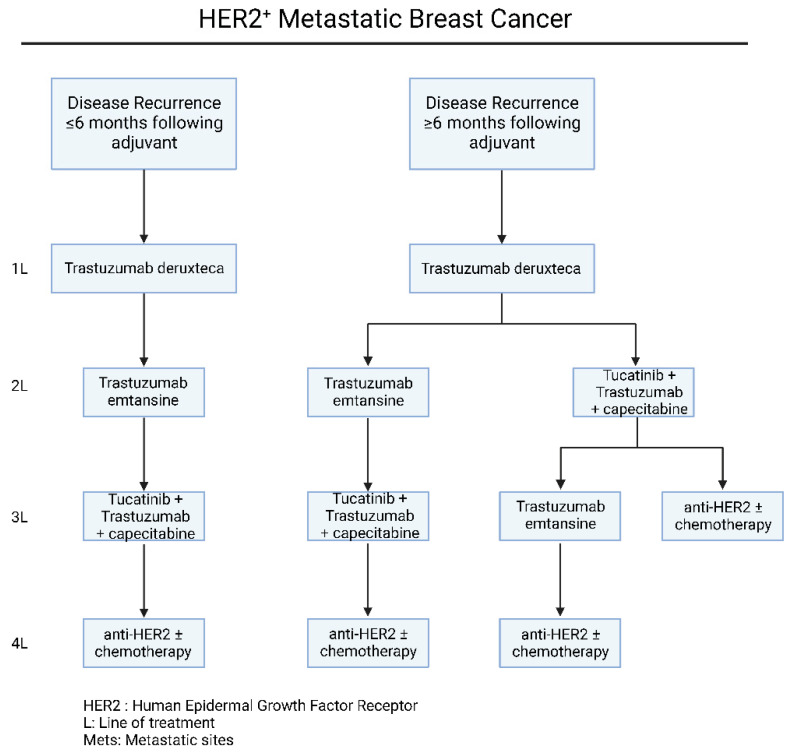
Current therapeutic algorithm for the management of HER-2^+^ mBC. Proposed therapeutic algorithm for patients with HER-2^+^ metastatic breast cancer [52]. The abbreviations of the terms used in the figure are outlined in the lower part of the algorithm.

As far as the therapeutic strategies of HER-2^+^ metastatic breast cancer are concerned, the main clinical factor that determines the first-line therapy option is the time of recurrence after adjuvant therapy (Figure 4). To begin with, based on recent guidelines and experts’ opinion, the combination of trastuzumab and pertuzumab with a single chemotherapeutic reagent, should be considered as the first-line of treatment in patients with recurrence after 6 months of adjuvant treatment [54] (Figure 4). The usage of pertuzumab with the widely used trastuzumab, has been validated through the large phase III CLEOPARTA trial, which compared the addition of pertuzumab versus placebo, in HER-2^+^ mBC patients that have received trastuzumab, and docetaxel [55,56]. Specifically for CLEOPATRA trial, the OS in the pertuzumab receiving group was 56.5 months (95% CI, 49.3 to not reached), compared to 40.8 months (95% CI, 35.8 to 48.3) in the group receiving the placebo combination (HR = 0.68; 95% CI, 0.56 to 0.84; *p* < 0.001) [55,56]. The therapeutic regimen of pertuzumab, is a monoclonal antibody that inhibits the dimerization of HER-2 by binding the extracellular domain II of the protein [57]. Due to its targeting of HER-2, the trastuzumab-pertuzumab combination provides a multi-level inhibition against these tumors, radically increasing therapeutic responses [58,59,60].

For patients that were presented with a recurrence in less than 6 months or progressed on trastuzumab and/or pertuzumab-based chemotherapy, the administration of T-DM1 should be considered as the second-line of choice (Figure 4). The FDA-approved T-DM1 regiment consists of the anti-HER-2 antibody trastuzumab, stably linked with microtubule-inhibitory agent DM1, in a 1:3.5 ratio [61,62]. This chemical structure allows specific drug delivery to HER2-overexpressing breast cancer cells intracellularly. The efficacy and safety profiling of T-DM1, is based on the results of EMILIA [61] and TH3RESA [62,63] phase III clinical trials, which compared T-DM1 with lapatinib plus capecitabine or chemotherapy plus trastuzumab, respectively.

Beyond targeted anti-HER-2 therapies, there are several drug regimens that have been FDA approved for patients that have progressed upon trastuzumab, pertuzumab and T-DM1. Nevertheless, there is no definite clinical algorithm for the management of these patients and the optimal sequence of drug administration remains largely unclear, depending mainly on the clinical characteristics, site of progression and toxicity profile. As far as these therapeutic regimens are concerned, tucatinib is a Tyrosine Kinase Inhibitor (TKI) with biochemical high specificity against HER-2 kinase domain [64]. The efficacy of tucatinib in combination with trastuzumab and capecitabine, was addressed in the phase II HER2CLIMB trial [65,66], leading to approval of this combination in 2020, for patients with advanced or metastatic HER-2^+^ mBC and have previously received anti-HER-2 based therapies. Notably, based on the results of this trial, on the arm of patients with brain metastasis, the 1-year PFS was 24.9%, compared to 0% in the placebo group [67,68], with subsequent increase in the reported quality of life [69], making this combination preferred for the brain metastatic disease (Figure 4). At this point, it is important to mention the recent developments in HER-2 low mBC. HER-2 low expression is generally defined as a IHC score of 1+ or as an IHC score of 2+ with negative results on in situ hybridization [68]. Based on the DESTINY-Breast04 clinical phase III trial, trastuzumab deruxtecan was compared with chemotherapy of physician’s choice. In this cohort, the PFS in the trastuzumab deruxtecan group was 9.9 months and 5.1 months in the physician’s choice group (HR = 0.50; *p* < 0.001), while the OS was 23.4 months and 16.8 months, respectively (HR = 0.64; *p* = 0.001) [69,70]. Based on these results, trastuzumab deruxtecan has been approved for the treatment of HER2-Low mBC. Furthermore, based on a recent clinical phase III trial, DESTINY-Breast03, trastuzumab deruxtecan achieved significantly longer progression free survival compared to trastuzumab emtansine (TDM-1) (HR = 0.55; 95% CI, 0.36 to 0.86), in HER-2^+^ mBC patient who progressed following treatment with anti-HER2 antibodies and a taxane [71,72].

Another FDA-approved oral TKI, neratinib, irreversibly inhibits HER-1, HER-2 and HER-4, promoting cell death through ferroptosis induction [73]. NALA phase III clinical trial addresses the combination of neratinib with capecitabine with lapatinib plus capecitabine [74,75]. Overall, the neratinib plus capecitabine treatment significantly prolonged PFS and reduced the percentage of patients with brain metastatic disease that required CNS intervention [74,75]. Based on these results, neratinib plus capecitabine combination is approved for patients with advanced or metastatic HER-2^+^ mBC after two or more anti-HER-2 lines of therapy. Nevertheless, neratinib was characterized from grade 3 diarrhea, even though the patients received mandatory anti-diarrheal prophylaxis during the study. More importantly, we need to mention that this clinical observation has been radically improved with the new dose escalation approaches, based on the CONTROL trial [76]. Last but not least, lapatinib is another FDA-approved oral TKI, reversibly inhibiting HER-1, HER-2 and EGFR. The results of a phase III clinical trial assessing the efficacy of lapatinib plus capecitabine compared to capecitabine alone, demonstrated that lapatinib treatment prolongs the progression interval, without increasing the observed side effects [77]. These results led to the FDA approval of lapatinib plus capecitabine for patients with HER-2^+^ mBC who had progressed upon treatment with anthracycline, taxanes, and trastuzumab (Figure 4).

### 4.3. Treatment of Triple Negative mBC

Compared to the two latter subtypes of breast cancer, Triple Negative Breast Cancer (TNBC) is characterized with significantly high risk of recurrence after treatment. Even though the majority of the patients presented with metastatic manifestations over the course of the disease in the past, in recent years the approval of new emerging therapies has significantly prolonged the survival and the pathological complete response (pCR) in this subgroup [78]. To begin with, we need to mention several recent landmark clinical trials that have shaped the clinical management of TNBC mBC. Firstly, based on the ASCENT clinical trials [79] for the treatment of TNBC mBC, patients were treated with sacituzumab govitecan versus single-agent chemotherapy of the physician’s choice (eribulin, vinorelbine, capecitabine, or gemcitabine). Sacituzumab govitecan is an antibody–drug conjugate composed of SN-38 (topoisomerase I inhibitor) and an antibody targeting the human trophoblast cell-surface antigen 2 (Trop-2), coupled through a linker. Based on this study, the median progression-free survival in patients treated with sacituzumab govitecan was 5.6 months (95% CI, 4.3 to 6.3) and 1.7 months (95% CI, 1.5 to 2.6) compared with those treated with chemotherapy alone (HR = 0.41; 95% CI, 0.32 to 0.52; *p* < 0.001) [79].

**Figure 5:**
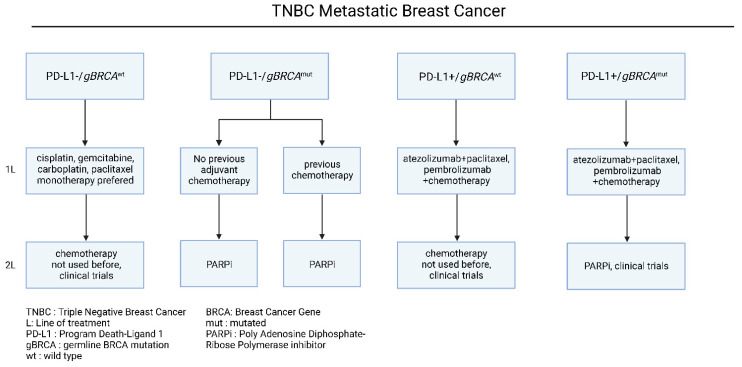
Current therapeutic algorithm for the management of TNBC mBC. Proposed therapeutic algorithm for patients with Triple Negative metastatic breast cancer [79,80]. The abbreviations of the terms used in the figure are outlined in the lower part of the algorithm.

Nevertheless, TNBC is also characterized by extensive chemo-sensitivity with high rates of pathological complete response after chemotherapy among the other breast cancer subtypes [51]. Based on recent advancements in molecular target identification, Programmed death-ligand 1 (PD-L1) and germline Breast Cancer gene (g*BRCA*) mutational status have been identified as main determinants of therapeutic approaches (Figure 5). To begin with, in patients with negative PD-L1 expression and wild type *BRCA* status, cytotoxic chemotherapy agents are considered the treatment of choice [80,81,82], especially in patients who have not received this chemotherapy class before [80,81,82]. Even though chemotherapy is associated with higher clinical response rates, and it is preferred in patients with extensive visceral disease, it has not been proved to prolong the overall and progression-free survival [49,50]. In patients who develop progression upon first-line treatment, it is recommended the administration of not previously used chemotherapy [82,83], or the enrollment in clinical trial protocols, a subject that will be expanded in a later section (Figure 5, Table 2 and Table 3).

In patients harboring germline *BRCA* mutations, the therapeutic approach includes the usage of platins-based chemotherapy and/or PARP inhibitors. The *BRCA* genes (*BRCA1*, *BRCA2*) encode proteins that participate in the DNA double-stranded breaks and homologous recombination, with their mutations to induce significant impairment in the DNA repair system [84]. On the one hand, platin-based chemotherapy introduces multiple single-stranded breaks in DNA, leading to synthetic lethality and apoptosis in g*BRCA*^mut^ tumors, due to their inability to repair DNA breaks [84]. On the other hand, Polyadenosine Diphosphate-Ribose Polymerase (PARP) complex maintains cellular homeostasis through a plethora of biological functions, that include the DNA repair system [85]. Similar to platins, PARP inhibitors interfere with the DNA damage response, leading to synthetic lethality in g*BRCA*^mut^ patients [86,87]. The effectiveness of platinum-based chemotherapy in HR^+^/HER-2^−^ and TNBC patients was proved in the TNT phase III clinical trial, in which carboplatin significantly enhanced the response rates (68% vs. 33%) and prolonged the PFS (6.8 vs. 4.4 months), compared to docetaxel [88]. In the case of PARP inhibitors, two large phase III clinical trials, namely the OLYMPIAD and EMBRACA studies, demonstrated significantly prolonged PFS in the PARP inhibitor group, compared to chemotherapy (7.0 vs. 4.2 months in OLYMPIAD and 8.6 vs. 5.6 months in EMBRACA) [12,89]. Notably, in both trials, PARP inhibition was associated with grade 3 hematological toxicities. These studies led to the FDA approval of talazoparib and olaparib for patients with g*BRCA*^mut^/HER-2^−^ metastatic breast cancer in 2018.

On the other hand, due to its unique biological background, TNBC is considered highly immunogenic, a characteristic linked with its high tumor mutational burden (TMB), among the other breast cancer subtypes [90]. To this extent, given that high TMB is associated with the generation of neoantigens and immune cell infiltration in the tumor-microenvironment [91], the effectiveness of immune checkpoint inhibitors in the clinical outcomes of TNBC patients has been previously investigated. In the large stage III clinical trial Impassion 130, the combination of nab-paclitaxel with atezolizumab was compared to nab-paclitaxel alone in patients with metastatic TNBC. Based on the results of this trial, the atezolizumab/nab-paclitaxel combination significantly prolonged the PFS compared to nab-paclitaxel alone (7.2 vs. 5.5 months, HR = 0.8, *p* = 0.002), without demonstrating any benefit in the OS (21.3 vs. 17.6 months, HR = 0.84, *p* = 0.08) [92]. Notably, specifically in the PD-L1^+^ patient subgroup, the investigated combination achieved prolonged PFS (7.5 vs. 5.0 months, HR = 0.62, *p* < 0.001) and OS (25.0 vs. 15.5 months, HR = 0.62, *p* < 0.001), compared to monotherapy, with parallel toxicity profiling [92,93]. It is important to mention that regardless of these results, the atezolizumab/nab-paclitaxel combination approval for metastatic TNBC has been withdrawn by the FDA. More importantly, according to the KEYNOTE-355 clinical phase III, the addition of pembrolizumab to chemotherapy led to significantly longer PFS than chemotherapy alone, in patients with PD-L1^+^ (CPS > 10) mBC TNBC (HR = 0.73; 95% CI, 0.55 to 0.95; *p* = 0.0185) [93,94]. Further clinical studies with a larger patient cohort are needed to address its effectiveness in PD-L1^+^ TNBC patients [94] (Figure 5). Given that TNBC has a higher frequency metastasizing in the brain, a summary of proposed therapeutic choices and indications for brain metastasis mBC are outlined in Table 2.

## 5. Emerging Therapies and Clinical Trials for mBC

**Figure 6:**
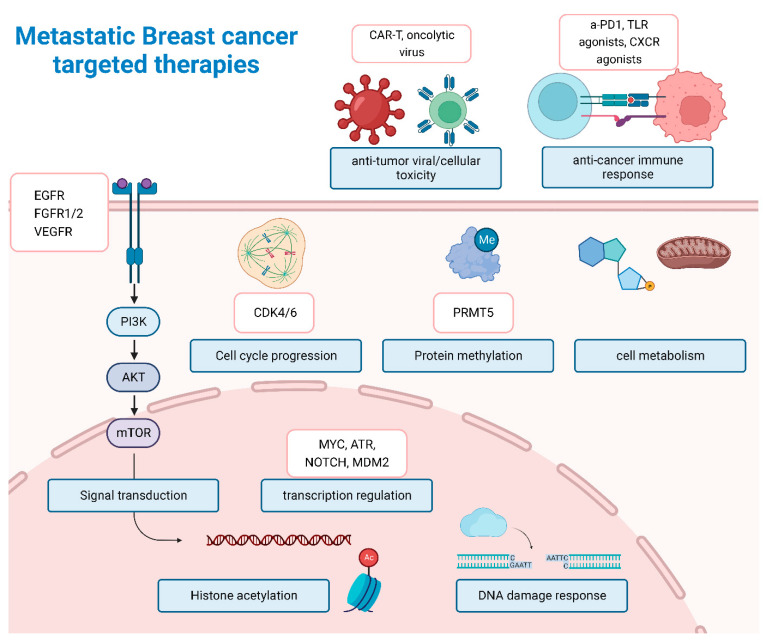
Overview of molecular targets of drugs under current clinical trials for BC. Drugs, pharmaceutical combinations and their targeted molecular pathways currently tested in clinical trials. Details on the clinical trials and abbreviation can be found in Table 3 and the corresponding section.

Approximately 70% of mBC are of luminal subtype. Based on this clinical phenomenon, our clinical efforts are focusing on endocrine-based therapies over the years, an approach that is inadequate to reverse the course of disease, with many patients developing resistance and disease progression [115]. The combination of identification of targetable mutations and classification of breast cancer subtypes allows for more individualized targeted therapies. [115] Ongoing clinical trials for mBC, as summarized in Table 3, focus on successfully targeting genes within signaling pathways, including a plethora of signaling, transcriptional and immune-related pathways (Figure 6). To begin with, Selective Estrogen Receptor Degrader (SERD), such as fulvestrant, exhibits tumor growth inhibition through binding to estrogen-receptors leading to complete anti-estrogen activity [116]. A new generation of SERDs are currently being tested in patients with metastatic HR^+^ breast cancer, as monotherapy or as a combination therapy [117] (Table 3, Figure 6). On the other hand, an alternative therapeutic strategy is the focus on the downstream activation of the PI3K-AKT-mTOR signaling pathway, known to be implicated in cancer proliferation, survival, and metastasis [118]. Activation of the PIK3 leads to recruitment of the AKT kinase and subsequently intracellular cascade of phosphorylation of mTOR, a potent driver of cancer cell progression and survival [118]. PI3K mutation and AKT activation are also paramount in endocrine therapy resistance [119]. To this extent, several ongoing clinical trials are investigating the efficacy and safety of PI3K/AKT/mTOR inhibitors, in combination with estrogen therapy and standard-of-care chemotherapy.

Another critical pathway involved in the endocrine resistance of mBC is cyclin D1 and cyclin-dependent kinases (CDKs). Based on past reports, the dysregulation of the cyclin D1/CDK4/6 pathway is crucial for cancer tumorigenesis as this is involved in cell cycle progression. [120] CDK4/6 inhibitors block the G1-to-S cycle transition in cancer cells leading to tumor growth control. [120] Based on ongoing clinical trials, clinical investigators are focusing on the therapeutic potential of CDK4/6i in combination with novel therapies, such as AKTi, Immunotherapy and new generation anti-HER2 antibodies (Table 3, Figure 6) [121]. Another metabolic pathway linked in endocrine resistance is the mevalonate pathway, primarily involved in the synthesis of cholesterol and isoprenoids. The output of this biological process is the generation of the 3-hydroxy-3-methyl-glutaryl-CoA reductase (HMGCR) that has been associated with cancer growth leading to poorer prognosis. [122] Hence, statins, the HMGCR inhibitors, are currently of increasing translational interest for inhibiting tumor growth and angiogenesis.

As outlined in preceding sections, compared to other mBC subtypes, TNBC, is characterized by a high immunogenic profile, increasing numbers of tumor-infiltrating lymphocytes and PD-L1 expression making it a suitable target for immunotherapy [123,124]. To this extent, due to tremendous advancements on the field of cancer immunology, over the last 5 years several research and translational groups have developed and are recently testing, a plethora of immuno-modulatory molecules, including novel anti-PDL1 antibodies, cytokine antagonists, immune receptor agonists, ex vivo-engineered dendritic cells and T-cells, mRNA vaccines and oncolytic viruses [124] (Table 3, Figure 6).

Last but not least, protein networks and protein-to-protein interactions have been extensively investigated and implicated as a milestone of cancer progression [125]. These protein networks and interactions include transcription factors, protein receptors, protein modifiers and repair enzymes. Among these categories, several protein-targeted inhibitors against the cellular signal transductors c-Myc, NOTCH, MDM2 and FGFR, the protein methyltransferase PRMT5, histone acetyltransferases (HDAC), and others (Table 3 Figure 6). One of the most translationally investigated families of inhibitors are the ones against Poly (ADP-ribose) polymerases (PARP), enzymes involved in DNA repair, with specific importance in TNBC with BRCA1/2 inactivation. Thus, in ongoing clinical trials, several novel combinations of PARPi with CDK4/6i, immunotherapy and/or targeted therapies, are currently being investigated for their clinical efficacy and their ability to induce synthetic lethality [126,127].

## 6. Conclusions/Future Directions

Metastatic breast cancer is a complex clinical condition, while being historically characterized by poor clinical outcomes. In this review, we collected evidence from tools used for the molecular classification of these tumors, along with impactful predictive and prognostic factors of the disease. More importantly, we outline that the classification of the molecular subtype of mBC is crucial for the proper therapeutic approach of each patient group, including HR^+^, HER-2^−^ and TN metastatic breast cancer. Due to recent molecular and translational advancements, the clinicians have a powerful arsenal of targeted therapeutic options to treat mBC, achieving long-lasting clinical outcomes, while improving the quality of life of these patients. In this review, we systematically outlined the recent clinical advancements, past clinical trials, the approved pharmacological combinations and guidelines for the therapeutic approach of mBC subgroups.

As we enter in the era of personalized and precision oncology, a plethora of new and in-depth studied classes of drugs are being currently tested in randomized clinical trials for their effectiveness in mBC. In our review, we captured the recent advancements and trends in the biomedical translational research around metastatic breast cancer. Future molecular and clinical studies need to identify new precision-medicine targets and pathways, while also addressing the optimal clinical subgroups that can benefit from novel therapeutic combinations and approaches. Collectively, our efforts should focus on ultimately transforming metastatic breast cancer, from a deadly consequence of breast cancer to a chronic disease, that women can live and thrive upon.

## Figures and Tables

**Table 1:** Prognostic and Predictive factors for metastatic Breast Cancer.

Prognostic Factors	Details	References
Relapse Free interval	≥2 years from primary breast cancer diagnosis is considered more favorable	Swenerton et al. [27] Hortobagyi et al. [28] Clark et al. [29] Harris et al. [30]
Metastatic sites: bones, chest wall, or lymph nodes	May have prolonged-free survival	Swenerton et al. [27] Hortobagyi et al. [28] Robertson et al. [31]
Metastatic sites: hepatic or lymphangitic pulmonary disease	Shorter PFS and OS	Barrios et al. [32]
Hormone receptor status	HR^+^: more favorable prognosis, ER^+^/PR^+^: significantly longer survival than single hormone receptor-positive tumors	Stuart-Harris et al. [33]
HER-2^+^ or TNBC	Shorter median survival	Clark et al. [29] Emi et al. [34] Ismail-Khan et al. [35]
PS (Performance Status)	Weight loss, high LDH and low PS are poor prognostic features	Swenerton et al. [27] Yamamoto et al. [36]
Circulating Tumor Cells (CTC) *	CTC ≥ 5/7.5 mL, poor prognosis with shortened PFS and OS	Bidard et al. [37] Smerage et al. [38]
Circulating tumor DNA (ctDNA) *	High ctDNA, increased risk of death	Ye et al. [39]

* CTC and ctDNA should not dictate treatment decisions. Abbreviations: PFS: Progression Free Survival; OS: Overall Survival; TNBC: Triple Negative Breast Cancer; LDH: Lactate dehydrogenase

**Table 2:** Summary of Brain Metastasis Treatment.

Indication	Therapy
Single, surgically accessible metastasis with favorable prognosis	Surgical resection [95,96,97,98,99,100] Whole brain radiotherapy (WBRT) [101]
Single, surgically inaccessible metastasis with favorable prognosis	Stereotactic Radiosurgery (SRS) with WBRT [102,103,104,105]
Multiple < 3 cm brain metastases, with favorable prognosis	SRS alone [106] Adjunctive WBRT [107]
Poor prognosis/PS	WBRT vs. SRS [108,109]
Patients with progressive extracranial disease or no feasible local therapy option	Systemic therapy based on subtypes [110]

**Table 3:** Summary of ongoing clinical trials for mBC derived from clinicaltrials.gov (accessed on 15 August 2022).

Subtype	Drug/Trial Name	Drug Target	Phase	HR (PFS/OS)	Trial Number/Status
HR^+^					
	Fulvestrant + AZD9496	SERD	I	-	NCT03236974 (completed) [111]
	Elacestrant (EMERALD)	SERD	III	-	NCT03778931 (ongoing)
	Giredestrant (GDC-9545) + Palbociclib	SERD	I	-	NCT03332797 (ongoing)
	Amcenestrant + fulvestrant	SERD	II	-	NCT04059484 (ongoing)
	Camizestrant (AZD9833)	SERD	II	-	NCT04214288 (ongoing)
	G1T48 + Palbociclib	SERD	I	-	NCT03455270 (ongoing)
	AC682	SERD	I	-	NCT05080842 (ongoing)
	H3B-6545	SERCA	I/II	-	NCT03250676 (ongoing)
	Atorvastatin (MASTER)	HMG-CoA reductase	III	-	NCT04601116 (ongoing)
	Onapristone + fulvestrant (SMILE)	Type I antiprogestin	II	-	NCT04738292 (ongoing)
	Hemay022 + endocrine therapy	Irreversible EGFR inhibitor	I	-	NCT03308201 (ongoing)
	ARV-471	PROTAC	I/II	-	NCT04072952 (ongoing)
	AZD5363 + fulvestrant	AKTi	I/II	-	NCT01992952 (ongoing)
	Ipatasertib (GDC-0068) + fulvestrant	AKTi	III	-	NCT04650581 (ongoing)
	HS-10352	PIK3-p110α	I	-	NCT04631835 (ongoing)
	Everolimus + Exemestane	mTORC1/2 inhibitor	II	-	NCT03312738 (ongoing)
	AZD2014 + Palbociclib	mTORC1/2 inhibitor	I	-	NCT02599714 (ongoing)
	Crizotinib + Fulvestrant	ALK/MET inhibitor	II	-	NCT03620643 (ongoing)
	Cabozantinib + Fulvestrant	VEGFR2, MET, RET inhibitor	II	-	NCT01441947 (ongoing)
	Bevacizumab + Ixabepilone	VEGF inhibitor	III	-	NCT00785291 (ongoing)
	Zilovertamab vedotin (MK-2140)	ROR1 inhibitor	II	-	NCT04504916 (ongoing)
	Infigratinib + Palbociclib + Fulvestrant	FGFRi + CDK4/6i	Ib	-	NCT04504331 (ongoing)
	Ε7090 + Fulvestrant	FGFRi	Ι	-	NCT04572295 (ongoing)
	Bortezomib + fulvestrant	Proteasome inhibitor	II	-	NCT01142401 (ongoing)
	trifluridine/tipiracil (TAS-102) (TIBET)	nucleoside analog plus thymidine phosphorylase inhibitor	II	-	NCT04489173 (ongoing)
	trastuzumab deruxtecan ** (Breast04)	ADC	III	-	NCT03734029 (ongoing)
	sacituzumab govitecan (TROPiCS-02)	ADC/Topo I	III	-	NCT03734029 (ongoing)
	Dato-DXd (TROPION-Breast01)	TROP2-directed ADC	III	-	NCT05104866 (ongoing)
	APG-2575 ± Palbociclib	Bcl-2 inhibitor	Ib/II	-	NCT04946864 (ongoing)
	ALRN-6924 + Paclitaxel	MDM2 inhibitor	I	-	NCT03725436 (ongoing)
	abemaciclib ^#^	CDK4/6i	retro multicenter [112]	PFS: 5.1 vs. 5.7 m, OS: 17.2 vs. 15.3 m	-
	Dalpiciclib (SHR6390)	CDK4/6i	I	-	NCT04236310 (ongoing)
	HRS8807 + SHR6390	CDK4/6i	I	-	NCT04993430 (ongoing)
	PRT2527	CDK9	I	-	NCT05159518 (ongoing)
	Pembrolizumab (KEYNOTE 028)	IO	Ib	ORR: 12%	NCT02054806 (completed) [113]
	Nivolumab + ipilimumab + Nab-paclitaxel	anti-PDL1 + anti-CTLA-4	I	-	NCT04132817 (ongoing)
	Avelumab + Palbociclib + Endocrine therapy	IO + CDK4/6i	II	-	NCT03573648 (ongoing)
	Durvalumab + Olaparib + fulvestrant	anti-PDL1 + PARPi	II	-	NCT04053322 (ongoing)
	Tucidinostat + Exemestane	HDAC inhibitor	II	-	NCT04465097 (ongoing)
	Vorinostat + Pembrolizumab	HDAC inhibitor + IO	II	-	NCT04190056 (ongoing)
	ESR1 peptide vaccine + GM-CSF	Vaccine	I	-	NCT04270149 (ongoing)
HER-2^+^					
	Tucatinib (HER2CLIMB)	anti-HER-2	III	HR 0.58/0.85	NCT02614794 (completed)
	MCLA-128 + trastuzumab	NRG1 fusion inhibitor	II	-	NCT03321981 (ongoing)
	Palbociclib + anti-HER-2 (PATINA)	CDK4/6i	III	-	NCT02947685 (ongoing)
	Alpelisib + anti-HER-2 (EPIK-B2)	PIK3α inhibitor	III	-	NCT04208178 (ongoing)
	GDC-0084 + trastuzumab	PIK3 inhibitor	II	-	NCT03765983 (ongoing)
	Copanlisib + trastuzumab	PIK3α inhibitor	I/II	-	NCT02705859 (ongoing)
	Gedatolisib + Herceptin	PIK3 inhibitor	II	-	NCT03698383 (ongoing)
	Ibrutinib + trastuzumab	BTK inhibitor	I/II	-	NCT03379428 (ongoing)
	Ceralasertib (DASH)	ATR inhibitor	I/II	-	NCT04704661 (ongoing)
	AUY922 + trastuzumab	HSP90 inhibitor	I/II	-	NCT01271920 (completed)
	Ganitumab (I-SPY)	IGF-1R inhibitor	I/II	-	NCT01042379 (ongoing)
	TVB-2640 + trastuzumab	FASN inhibitor	II	-	NCT03179904 (ongoing)
	ladiratuzumab vedotin + trastuzumab	zinc transporter LIV-1 inhibitor	I	-	NCT01969643 (ongoing)
	DC1 (Dendritic Cell)-WOKVAC	Vaccine	II	-	NCT03384914 (ongoing)
	TPIV100	anti-HER- 2 Vaccine	II	-	NCT04197687 (ongoing)
	pNGVL3-hICD	anti-HER- 2 Vaccine	I	-	NCT00436254 (ongoing)
	KN035 + trastuzumab	Single Domain a-PD-L1	I/II	-	NCT04034823 (ongoing)
	M7824	PD-L1/TGFβ fusion protein	II	-	NCT03620201 (ongoing)
	PRS-343 + atezolizumab	4-1BB Ab	Ib	-	NCT03650348 (ongoing)
	SBT6050 + anti-HER-2	TLR8 agonist	I/II	-	NCT05091528 (ongoing)
	BPX-603	CAR-T cells	I/II	-	NCT04650451 (ongoing)
TNBC					
	Goserelin	GnRH analog	II	-	NCT03444025 (ongoing)
	Nadunolimab + chemo	IL1RAP	I/II	-	NCT05181462 (ongoing)
	SKB264	TROP2-directed ADC	III	-	NCT05347134 (ongoing)
	ASTX660 + pembrolizumab (ASTEROID)	IAPi + IO	I	-	NCT05082259 (ongoing)
	OMO-103	anti-Myc CPP	I/II	-	NCT04808362 (ongoing)
	SKL27969	PRMT5	I/II	-	NCT05388435 (ongoing)
	LY3023414 + Prexasertib	PIK3/AKT + CHEK1i	II	-	NCT04032080 (ongoing)
	Sitravatinib	Multi-kinase inhibitor	II	-	NCT04123704 (ongoing)
	Tak-228 + Tak-117 + Chemo	PIK3/AKT/mTORC1i	II	-	NCT03193853 (ongoing)
	Eganelisib + pembrolizumab + bevacizumab + paclitaxel	PIK3/AKT/mTORC1i + IO + anti-VEGF	I/II	-	NCT05390710 (ongoing)
	Capivasertib + Paclitaxel (CAPItello-290)	pan-AKTi + Chemo	III	-	NCT03997123 (ongoing)
	Gedatolisib + Talazoparib	PIK3i + PAPRi	I/II	-	NCT03911973 (ongoing)
	AZD6738 + Olaparib + Durvalumab (PHOENIX)	ATRi + PARPi + IO	II	-	NCT03740893 (ongoing)
	Olinvacimab + pembrolizumab	anti-VEGFR2 + IO	II	-	NCT04986852 (ongoing)
	PMD-026	RSKi	I	-	NCT04115306 (ongoing)
	Talazoparib + Selinexor (START)	PARPi + XPO1i		-	NCT05035745 (ongoing)
	Chiauranib + capecitabine	Multi-kinase inhibitor	II		NCT05336721 (ongoing)
	TT-00420	Multi-kinase inhibitor	I		NCT03654547 (ongoing)
	AL101	γ-secretase NOTCHi	II	-	NCT04461600 (ongoing)
	ZEN003694 + Talazoparib	BET domain inhibitor + PARPi	II	-	NCT03901469 (ongoing)
	Binimetinib + Palbociclib	MEK1/2i + CDK4/6i	I/II	-	NCT04494958 (ongoing)
	Trilaciclib + Sacituzumab Govitecan	CDK4/6i + TROP-2 directed ADC	II	-	NCT05113966 (ongoing)
	Chidamide + chemo	HDAC	II/III	-	NCT04582955 (ongoing)
	Eryaspase + chemotherapy (TRYbeCA-2)	L-asparaginase	II/III	-	NCT03674242 (ongoing)
	Deferoxamine + chemo	Iron Binding agent	II	-	NCT05300958 (ongoing)
	SG001 + paclitaxel	IO	II	-	NCT05068141 (ongoing)
	Serplulimab + chemo	IO	III	-	NCT04301739 (ongoing)
	KN046 + paclitaxel	anti-PD-L1/CTLA-4	I/II	-	NCT03872791 (ongoing)
	CDX-1140 + CDX-301 + PLD Chemotherapy	CD40 agonist + anti-FLT3	I	-	NCT05029999 (ongoing)
	Romidepsin + nivolumab + cisplatin	HDAC + IO	I/II	-	NCT02393794 (ongoing)
	Tiragolumab + Atezolizumab + paclitaxel	anti-TIGIT + IO	I	-	NCT04584112 (ongoing)
	Fruquintinib +	anti-VEGF + IO	I/II	-	NCT04577963 (ongoing)
	Anlotinib + Tislelizumab	anti-VEGF/MEK + IO	II	-	NCT04914390 (ongoing)
	Niraparib + Dostarlimab + RT	PARPi + IO + RT	II	-	NCT04837209 (ongoing)
	Ipatasertib + Atezolizumab	AKTi + IO	III	-	NCT04177108 (ongoing)
	Magrolimab + Paclitaxel + Sacituzumab Govitecan	anti-CD47 + ADC	II	-	NCT04958785 (ongoing)
	CMP-001 + RT	TLR9 pDC agonist	II	-	NCT04807192 (ongoing)
	TIL LN-145	Tumor Infiltrating Lymphocytes	II	-	NCT04111510 (ongoing)
	BDB001 + atezolizumab + RT (AGADIR)	TRL7 agonist + IO	II	-	NCT03915678 (ongoing)
	Spartalizumab LAG525 + NIR178 + capmatinib	A2AR antagonist + METi+ IO	I	-	NCT03742349 (ongoing)
	Sitravatinib + Tislelizumab	Multi-kinase inhibitor + IO	II	-	NCT04734262 (ongoing)
	Ivermectin + pembrolizumab	IMPα/β1 stabilizer + IO	II	-	NCT05318469 (ongoing)
	Tavokinogene telseplasmid + pembrolizumab	IL-12 injecting tele- monitored plasmid + IO			NCT03567720 (ongoing)
	CF33-hNIS-	Oncolytic Virus-	I	-	NCT05081492
	antiPDL1	conjugated with IO			(ongoing)
	RBX7455	Microbiota-based formulation	I	-	NCT04139993 (ongoing)
	ADV/HSV-tk + RT + Pembrolizumab	Oncolytic Virus + RT + IO	II	-	NCT03004183 (ongoing)
	mRNA-275 + Durvalumab	mRNA + IO	I	-	NCT03739931 (ongoing)
	PVX-410 + pembrolizumab + chemo	Vaccine + IO	II	-	NCT04634747 (ongoing)
	AE37 + pembrolizumab	Vaccine + IO	II	-	NCT04024800 (ongoing)
	X4P-001 + Toripalimab	CXCR4 antagonist + IO	I/II	-	NCT05103917 (ongoing)
	EGFR/B7H3 CAR-T	CAR-T cells	I	-	NCT05341492 (ongoing)
All subtypes	IO-based combinations	ADC, HDAC, anti-VEGF, CDK4/6i, PARP	I-III	-	Extensively reviewed [114]

** For HR+/HER-2 low-expressing mBC. # CDK4/6i was given after disease progression.

## Data Availability

All the data used for this study are included in the manuscript.

## Abbreviations

PFS: Progression Free Survival; OS: Overall Survival; SERD: selective estrogen receptor degrader or down regulator; SERCA: selective estrogen receptor alpha covalent antagonist; HMG-CoA: 3-hydroxy-3-methylglutaryl coenzyme A; EGFR: Epidermal Growth Factor Receptor; PROTAC: ER proteolysis–targeting chimeras; PI3K: Phosphatidylinositol-4,5-bisphosphate 3-kinase; mTORC: mammalian target of rapamycin complex; ALK: Anaplastic lymphoma kinase; MET: mesenchymal epithelial transition factor; VEGF: Vascular endothelial Growth Factor; RET: Rearranged during Transfection; ROR1: Receptor Tyrosine Kinase Like Orphan Receptor 1; FGFR: Fibroblast growth factor receptor; ADC: Antibody-drug conjugates; Topo: Topoisomerase; TROP-2: Trophoblast cell-surface antigen-2; Bcl2: B-cell lymphoma 2; MDM2: mouse double minute 2; CDK: Cyclin Dependent Kinase; IO: immunotherapy; ORR: Overall Response Rate; PD-L1: Program Death Ligand 1; CTLA-4: cytotoxic T-lymphocyte-associated antigen 4; PAPR: poly ADP-ribose polymerase; HDAC: Histone Deacetylase; GM-CSF: Granulocyte-macrophage colony-stimulating factor; NRG1: Neuregulin 1; BTK: Bruton Tyrosine Kinase; ATR: ataxia telangiectasia and Rad3-related protein; Hsp90: heat shock protein 90; IGF-1R: Insulin-like Growth Factor-1 Receptor; FASN: Fatty Acid Synthase; TGFβ: Transformation Growth Factor beta; CAR-T: Chimeric antigen receptor T cells; GnRH: Gonadotropin-releasing hormone; IL1RAP: interleukin-1 receptor accessory protein; IAP: Inhibitors of Apoptosis Proteins; CPP: Cell Penetrated Peptide; PRMT5: Protein Arginine Methyltransferase 5; CHEK1: Checkpoint kinase 1; RSK: P90 ribosomal S6 kinase; XPO1: Exportin 1; BET: Bromo- and Extra-Terminal domain; MEK: Mitogen-activated protein kinase kinase, RT: radiotherapy, FLT3: fms-like tyrosine kinase 3; PLD: Pegylated liposomal doxorubicin; TIGIT: T cell immunoreceptor with Ig and ITIM domains; TLR: Toll-Like Receptor; DC: Dendritic Cells; A2AR: adenosine 2A receptor; IMPα/β1: Importin alpha/beta 1 CXCR4: C-X-C Motif Chemokine Receptor 4. ***** Hazard ratio for OS and PFS or OS/PFS if reported in the study. ** For HR^+^/HER-2 low-expressing mBC. ^#^ CDK4/6i was given after disease progression.
